# microRNA Profiles in Parkinson's Disease Prefrontal Cortex

**DOI:** 10.3389/fnagi.2016.00036

**Published:** 2016-03-01

**Authors:** Andrew G. Hoss, Adam Labadorf, Thomas G. Beach, Jeanne C. Latourelle, Richard H. Myers

**Affiliations:** ^1^Department of Neurology, Boston University School of MedicineBoston, MA, USA; ^2^Graduate Program in Genetics and Genomics, Boston University School of MedicineBoston, MA, USA; ^3^Bioinformatics Program, Boston UniversityBoston, MA, USA; ^4^Banner Sun Health Research InstituteSun City, AZ, USA; ^5^Genome Science Institute, Boston University School of MedicineBoston, MA, USA

**Keywords:** Parkinson's disease, miRNA, small RNA sequencing, dementia, cortex, Huntington's disease

## Abstract

**Objective:** The goal of this study was to compare the microRNA (miRNA) profile of Parkinson's disease (PD) frontal cortex with normal control brain, allowing for the identification of PD specific signatures as well as study the disease-related phenotypes of onset age and dementia.

**Methods:** Small RNA sequence analysis was performed from prefrontal cortex for 29 PD samples and 33 control samples. After sample QC, normalization and batch correction, linear regression was employed to identify miRNAs altered in PD, and a PD classifier was developed using weighted voting class prediction. The relationship of miRNA levels to onset age and PD with dementia (PDD) was also characterized in case-only analyses.

**Results:** One twenty five miRNAs were differentially expressed in PD at a genome-wide level of significance (FDR *q* < 0.05). A set of 29 miRNAs classified PD from non-diseased brain (93.9% specificity, 96.6% sensitivity). The majority of differentially expressed miRNAs (105/125) showed an ordinal relationship from control, to PD without dementia (PDN), to PDD. Among PD brains, 36 miRNAs classified PDD from PDN (sensitivity = 81.2%, specificity = 88.9%). Among differentially expressed miRNAs, miR-10b-5p had a positive association with onset age (*q* = 4.7e-2).

**Conclusions:** Based on cortical miRNA levels, PD brains were accurately classified from non-diseased brains. Additionally, the PDD miRNA profile exhibited a more severe pattern of alteration among those differentially expressed in PD. To evaluate the clinical utility of miRNAs as potential clinical biomarkers, further characterization and testing of brain-related miRNA alterations in peripheral biofluids is warranted.

## Introduction

Parkinson's disease (PD) is a progressive movement disorder, characterized clinically by resting tremor, rigidity, bradykinesia and postural instability (Parkinson, 2002). Motor symptoms are accompanied by the loss of dopamine-producing neurons in the *substantia nigra pars compacta*, and associated with widespread deposition of cytoplasmic protein inclusions, largely composed of insoluble α-synuclein throughout the brain (Spillantini et al., 1998).

While neuropathological hallmarks contribute to the degeneration of the nigrostriatal dopaminergic system, the etiology, clinical heterogeneity and fundamental pathological mechanisms by which preferential neuronal loss occurs in PD are largely unknown. Monogenic forms of PD represent only a minority of cases (Hamza and Payami, 2010; Barrett et al., 2015). The clinical manifestations of Parkinson's disease (PD) are highly heterogeneous. Approximately one-third of patients experience dementia, which has significant ramifications for quality of life and burden of care (Breteler et al., 1995; Edwards et al., 2010). Additionally, there is wide variation in the age of motor onset (ranging from age 20 to 90; Schrag et al., 1998), with young-onset (before age 50) and representing 5–10% of PD cases (Golbe, 1991).

The microRNA (miRNA) profile of PD brains may offer insight into the molecular and pathological mechanisms that occur in the disease. miRNAs are short, noncoding RNAs that inhibit translation through sequence-specific binding of the 3′-untranslated region of target messenger RNAs. miRNAs post-transcriptionally regulate a set or multiple sets of genes simultaneously, and in the brain, their regulatory effects have profound effects on neuronal development, differentiation and maturation (O'Carroll and Schaefer, 2013). Deregulation of miRNAs has been implicated in neurodegenerative diseases (Chan and Kocerha, 2012), and several studies suggest miRNAs may impact PD pathogenesis (Kim et al., 2007; Gehrke et al., 2010).

Although the substantia nigra (SN) is the most affected brain region in PD, as many as 80% of dopaminergic neurons are lost by the time of death (Cheng et al., 2010). Consequently, expression analysis comparing PD to normal SN from post-mortem brains may only highlight miRNAs associated with changes in cellular composition rather than miRNAs related to the disease process itself. For this study, we performed miRNA sequence analysis of 29 PD and 33 neuropathologically-normal control from post-mortem prefrontal cortex, a region which contains both dopaminergic neuron projections and pathological hallmarks (Beach et al., 2009; Ferrer et al., 2011), but does not exhibit the extent of cell death observed in SN.

Sequence analysis allows for high-resolution, genome-wide quantification of miRNA molecule abundance. We compared miRNA levels between PD and controls to identify miRNA alterations and based on their miRNA profile, classified PD from non-diseased brains. Among the PD brains, we identified miRNAs associated with the presence or absence of dementia and age of motor onset, as well as identified a set of miRNAs altered in both PD and Huntington's disease, which may be relevant to the pathological processes that occur broadly across age related neurodegenerative diseases.

## Methods

### Sample information and small RNA sequencing

Frozen brain tissue from prefrontal cortex Brodmann Area 9 (BA9) for 29 PD samples and 33 control samples was obtained from the National Brain and Tissue Resource for Parkinson's Disease and Related Disorders at Banner Sun Health Research Institute, Sun City, Arizona (Beach et al., 2008), Harvard Brain and Tissue Resource Center McLean Hospital Belmont MA, and Human Brain and Spinal Fluid Resource Center VA West Los Angeles Healthcare Center, Los Angeles, CA. PD samples had insufficient Alzheimer's disease histopathology to quality for National Institute of Aging/Reagan “intermediate” or “high” probability (Hyman and Trojanowski, 1997). Sample information can be found in Table S1 and summarized in Table 1. The medical charts of all 29 PD samples were reviewed to obtain information regarding clinical diagnoses of dementia (PDD, *n* = 11), no evidence of dementia (PDN, *n* = 18). Twenty one subjects had information on the age of onset of motor symptoms. Family history information regarding PD was not available for these samples, but all samples were negative for the common known PD mutations in GBA and LRRK2. All samples were male. Agilent's BioAnalyzer 2100 system (or TapeStation 2200 equivalent) was used to determine RNA Integrity Number for RNA quality assessment. Differences in covariates were tested assuming unequal variance. No difference in postmortem interval (*p*-value = 0.10) or RNA integrity number (*p*-value = 0.08) was observed between PD and controls. PD and controls differed in ages at death (PD mean age = 77.6, control mean age = 68.1; *p*-value = 3.2e-3). Potential confounding by age at death was assessed in subsequent analyses. PDN and PDD samples did not differ in postmortem interval (*p*-value = 0.62), RNA integrity number (*p*-value = 0.27), age at onset (*p*-value = 0.24), duration (*p*-value = 0.44), or age at death (*p*-value = 0.28).

**Table 1:** **Summarized sample information**.

**Type**	***N***	**Motor onset**	**Disease duration**	**Age at death**	**Post mortem interval**	**RNA integrity number**
Control	33	–	–	68.1 ± 14.8	15.0 ± 8.7	7.6 ± 0.7
All diagnosed PD	29	66.5 ± 9.8	10.5 ± 6.5	77.6 ± 9.0^**^	11.1 ± 9.7	7.3 ± 0.7
PD, Non demented	18	64.1 ± 7.2	11.5 ± 6.4	76.1 ± 8.9	11.9 ± 9.2	7.2 ± 0.8
PD with dementia	11	69.8 ± 12.2	9.2 ± 6.7	79.9 ± 9.0	9.9 ± 10.9	7.5 ± 0.5

Ages at death were significantly different between controls and PD, as indicated (

** *p < 0.01 compared to controls). No differences between PDN and PDD were observed*.

Total RNA was isolated using QIAzol Lysis Reagent and purified using miRNeasy MinElute Cleanup columns. Samples were prepared using Illumina's TruSeq Small RNA Sample Prep Kit, according to the manufacturer's protocol, and sequenced on Illumina's HiSeq 2000 system with 1x51nt single-end reads at Tufts University and the Michigan State sequencing core facility.

### Statistical analysis

Reads were processed and counted as described previously (Hoss et al., 2015). R version 3.1.0, Bioconductor version 2.1.4, and DESeq2 version 1.40.0 were used for variance stabilizing transformation (VST) of count data. Batch correction was applied using ComBat using sva 3.10 (Johnson et al., 2007) and LIMMA version 3.20.8 (Smyth, 2005) was used for differential expression analysis of PD cases and controls. All PD samples were from a single batch, therefore DESeq2 normalized, VST counts from PD samples without batch correction were used for the PD-only analyses relating miRNA levels to clinical features. Differential expression analysis was performed using LIMMA twice, once including age of death as a covariate and once unadjusted. The results of the models were compared to determine if confounding by age of death (>10% effect change) was present. To further assess any residual confounding, regression analysis was performed, stratified by age. A cutoff based on the average age of all samples (72.5 years) was used to produce two groups of samples—one that contained 21 controls and 8 PD younger than 72.5 years and another that contained 12 controls and 21 PD older than 72.5 years of age. After multiple comparisons correction using a false discovery rate (Benjamini and Hochberg, 1995), FDR-adjusted *q*-values < 0.05 were reported as significant.

To further evaluate the differential expression patterns between PD cases and controls, unsupervised, Ward hierarchical clustering by Euclidian distance was applied using the heatmap2 function in the gplots R package (Warnes et al., 2015). Supervised, predictive modeling of case status was performed using the GenePatterns WeightedXVoting module using 29 PD-associated miRNAs with large effects (Reich et al., 2006).

To determine if miRNAs were associated with PD in the presence or absence of dementia, VST counts for PDN and PDD were compared using LIMMA, adjusting for age at death, and *p*-values were FDR-adjusted for the number of comparisons. miRNAs nominally associated with PDD were used to classify PD patients with and without dementia, as described above, using the weighted voting method. To determine beta estimates relative to control samples, PDN and PDD were separately compared to controls using LIMMA. In addition, cumulative logit models using the “ordinal” package in R (Christensen, 2015), were applied to test whether an ordinal relationship existed across control, PDN and PDD samples. FDR-corrected Chisq likelihood-ratio tests were used to determine significant miRNA associations.

Linear models were used to model the relationship between age of motor onset and miRNA levels among the 21 PD samples with onset data. Tests were performed genome-wide and exclusively among the set of differentially expressed miRNAs. FDR-adjusted *q*-values < 0.05 were reported. Models were run with and without adjustment for age at death.

Finally, to determine whether overlap in miRNA alterations exist between PD and HD brain, results from PD differential expression analysis were compared to those of our previously published Huntington's disease (HD) study (Hoss et al., 2015), which contain the same control brains. HD data was accessed from NCBI's Gene Expression Omnibus, series accession number GSE64977 (http://www.ncbi.nlm.nih.gov/geo/query/acc.cgi?acc=GSE64977), and analyzed using the same bioinformatics approach for differential expression analysis as described above (Bioconductor version 2.1.4, DESeq2 version 1.40.0, ComBat sva 3.10, LIMMA version 3.20.8). The probability of overlap of differentially expressed miRNA between the two diseases was assessed using a hypergeometric test (phyper function in the R package “stats”).

The miRNA sequence data analyzed here can be accessed from NCBI's Gene Expression Omnibus, series accession number GSE72962 (http://www.ncbi.nlm.nih.gov/geo/query/acc.cgi?acc=GSE72962).

## Results

### miRNA levels are altered in Parkinson's disease compared to non-disease brains

To identify miRNA differences in PD vs. non-disease subjects, miRNA sequence analysis was performed in prefrontal cortex (Brodmann Area 9) for 33 controls and 29 idiopathic PD samples (see Table 1). Results of differential expression analyses, correcting for sequencing batch effects and with and without adjustment for age at death are shown in Table S2. 125 miRNAs were significantly altered in PD after adjusting for age at death (FDR *q*-value < 0.05, see Table S2). Unadjusted results were similar, but confounding by age of death was observed for some miRNA, so adjusted results are reported here. Stratified analyses did not show evidence of confounding by age driving the results of the 125 reported miRNA. Most miRNA alterations were moderate, with 77% of the differentially abundant miRNAs (96/125) within a ±0.5 log fold change (LFC). The levels of 64 miRNAs were down-regulated, whereas the levels of 61 miRNAs were up-regulated in PD relative to controls.

**Figure 1:**
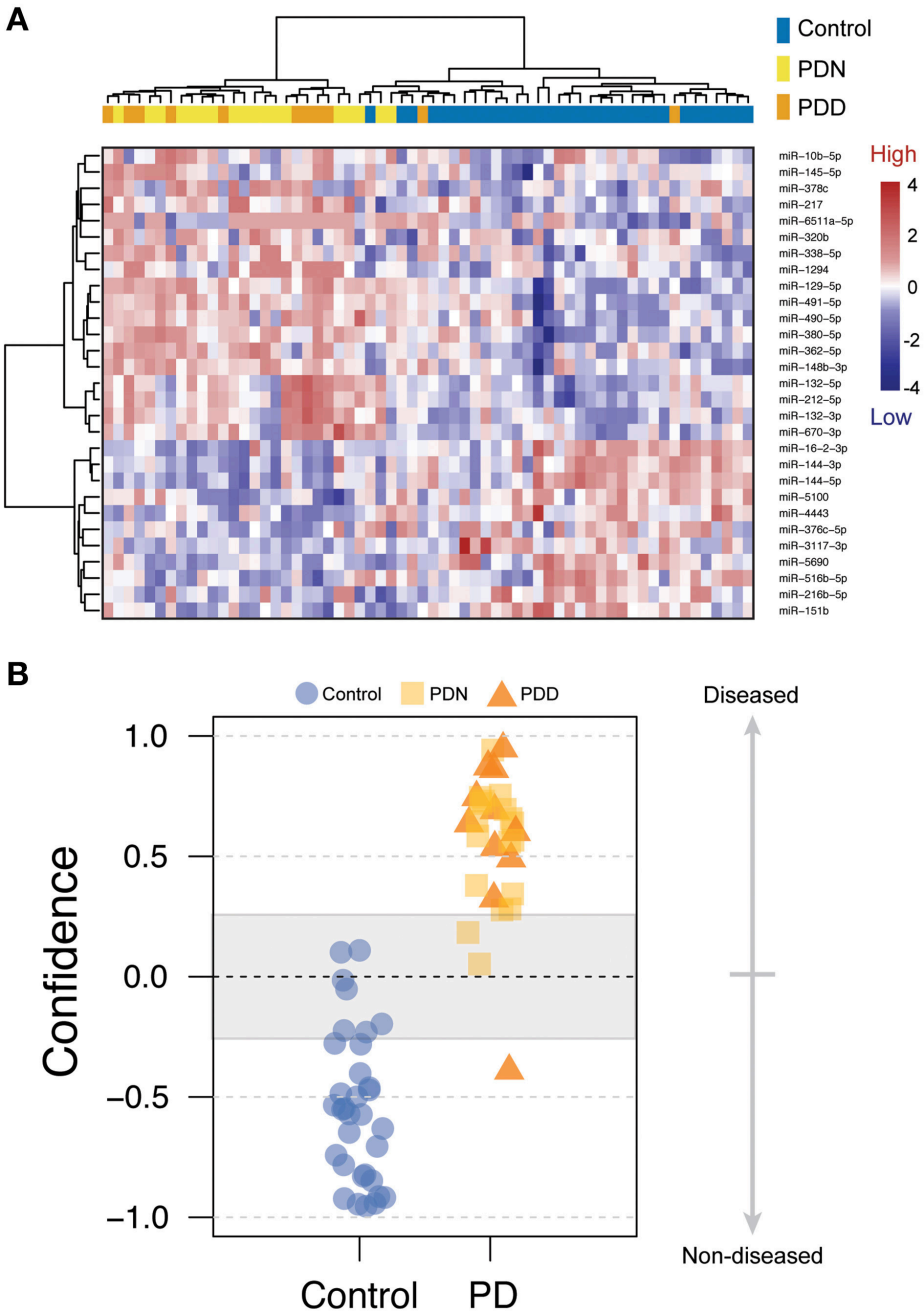
**miRNA changes related to Parkinson's disease**. **(A)** Heatmap of 29 miRNAs differentially expressed between PD and control prefrontal cortex samples with log fold changes (LFC) greater than 0.5 or less than −0.5. Scaled level values are color-coded according to the legend on the right. The dendrogram on the left depicts hierarchical clustering based on level. The top dendrogram depicts clustering based on the miRNA signal from each sample. The top bar indicates disease status [blue: control, yellow: PD, non-demented (PDN), orange: PD with dementia (PDD)]. **(B)** Disease prediction using 29 miRNAs. Scores less than zero were called as non-diseased whereas scores above zero were called as PD. Blue circles = control, yellow boxes = PD, orange triangles = PDN.

We used classification models to determine whether the levels of PD-related miRNAs in brain could accurately assign disease status. To select the most informative miRNAs, we filtered on effect size (LFC > 0.5 or LFC < −0.5). After filtering, 29 PD-related miRNAs were used in an unsupervised hierarchical cluster analysis. Samples clustered based on disease status with the exception of five PD which clustered with the controls, (see Figure 1A). To further assess whether miRNA levels could differentiate PD and control samples, disease status was predicted using a weighted voting classification with leave-one-out cross-validation. This model is internally tested by iteratively leaving one sample out, creating a training model by assigning a weighted linear combination based on the levels of the 29 miRNAs, and testing this model on the left out sample. Here, only three errors were observed using 29 miRNAs (two Type I errors, one Type II error), with 93.9% specificity, 96.6% sensitivity and an absolute error rate of 4.8% (see Figure 1B). Both Type I errors were called with low confidence (9.8–10.1%).

### miRNA alterations in Parkinson's disease with dementia

To assess whether miRNA differences specific to the PDD subtype were distinguishable from a generalized PD response, we performed a differential expression analysis comparing PDN to PDD using normalized VST count data from 18 PDN and 11 PDD samples. We observed no genome-wide significant (*q* < 0.05) miRNAs associated with dementia in PD, with or without adjustment for age or disease duration, after multiple correction testing (see Table S2). Even when limiting to the 125 differential expressed miRNAs we saw no significant differences between PDN and PDD. We however noted stronger directions of effect in PDD when separately comparing PDN vs. control and PDD vs. control, suggesting PDD may represent a more severe version of the PD miRNA profile spectrum.

**Figure 2:**
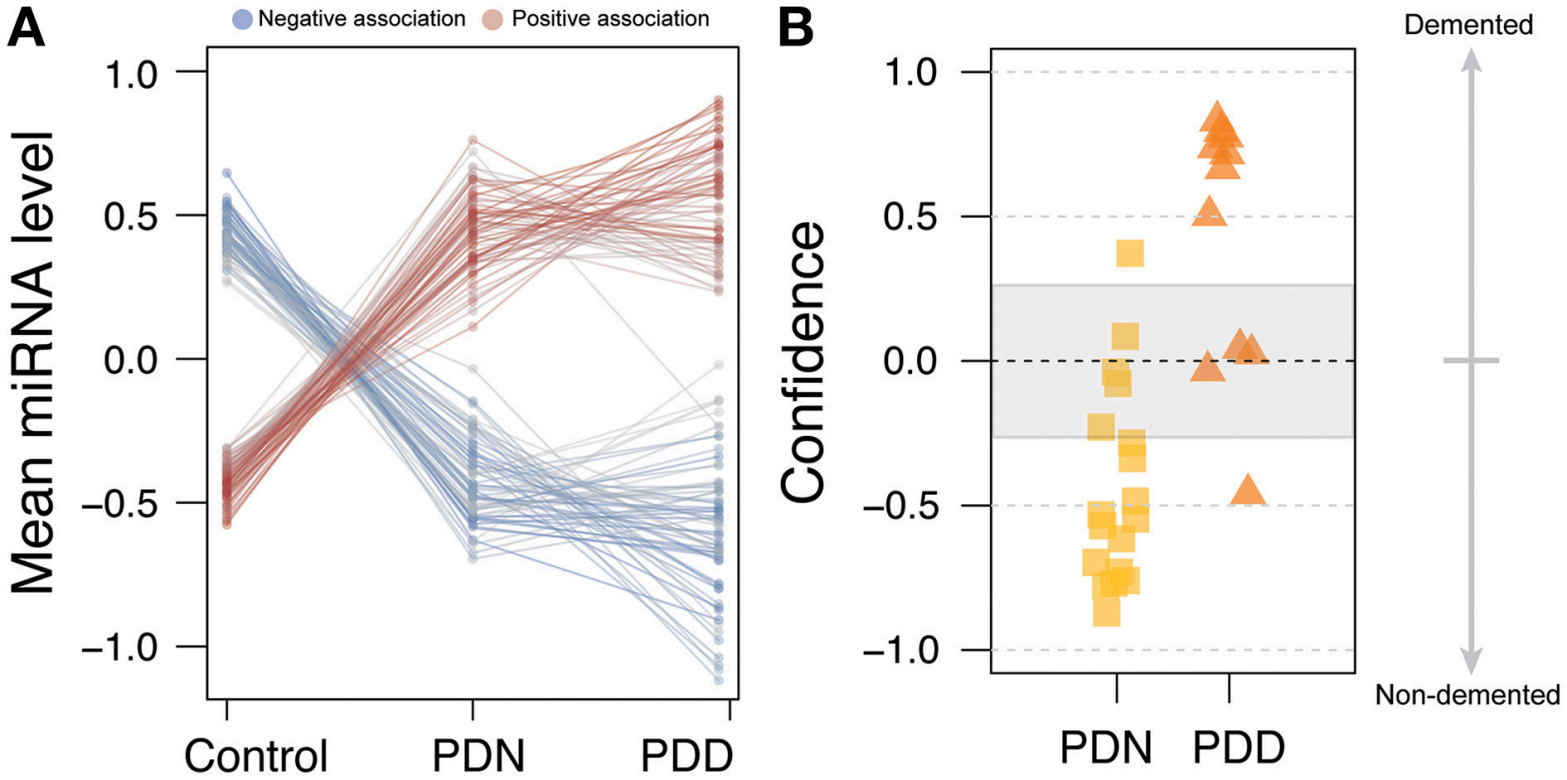
**miRNA profile for Parkinson's disease with dementia. (A)** Line plot for the 125 DE miRNAs in PD. The counts were scaled using Z-transformation, and the means were calculated for each miRNA for each condition (control, PDN, PDD). The line colors correspond to the beta estimates from the ordinal regression analysis, where blue=negative ordered relationship and red=positive ordered relationship. **(B)** PDN/PDD class prediction, using the 36 nominally significant miRNAs from PDN/PDD differential expression analysis. Four errors were observed among the 29 samples studied which may reflect heterogeneity in the etiology of PD.

To test whether PDD had increased miRNA alterations in comparison to PDN, we created an ordered categorical variable (controls, PDN, and PDD) and tested the association of this variable to genome-wide miRNA levels. 105 of the 125 differentially expressed miRNAs had a significant ordinal association (*q* < 0.05; see Table S2, Figure 2A), indicating that in the majority of differentially expressed miRNAs in PD, PDD samples exhibit larger differences than PDN cases as compared to controls for the same miRNAs.

We further investigated the clinical utility of these miRNA profiles for the assessment of dementia using classification analyses (WeightedXVoting; Reich et al., 2006). The 36 nominally significant miRNAs (*p* < 0.05) identified in the PDN/PDD comparison from LIMMA were used to classify disease state (See Figure 2B), though with more limited accuracy than the PD-control model (absolute error rate = 13.8%, sensitivity = 81.2%, specificity = 88.9%). Four miRNA features overlap between the control/PD and PDN/PDD models (miR-132-3p, hsa-miR-132-5p, hsa-miR-145-5p, hsa-miR-212-5p).

### miR-10b-5p levels are associated with the onset of motor symptoms in both Parkinson's and Huntington's disease

To understand whether deregulated miRNAs were specific to PD, or a general response to the neurodegenerative process, we compared miRNA that were significantly altered in PD to those significantly altered in Huntington's disease (HD). Twenty one miRNAs were found differentially expressed in both PD and HD experiments and the probability of this overlap occurring was more than that expected by chance (*p* = 5.4e-3). Importantly, of these 21 miRNAs, only two miRNAs had opposite directions of effect between diseases (miR-10b-5p, miR-320b).

**Figure 3:**
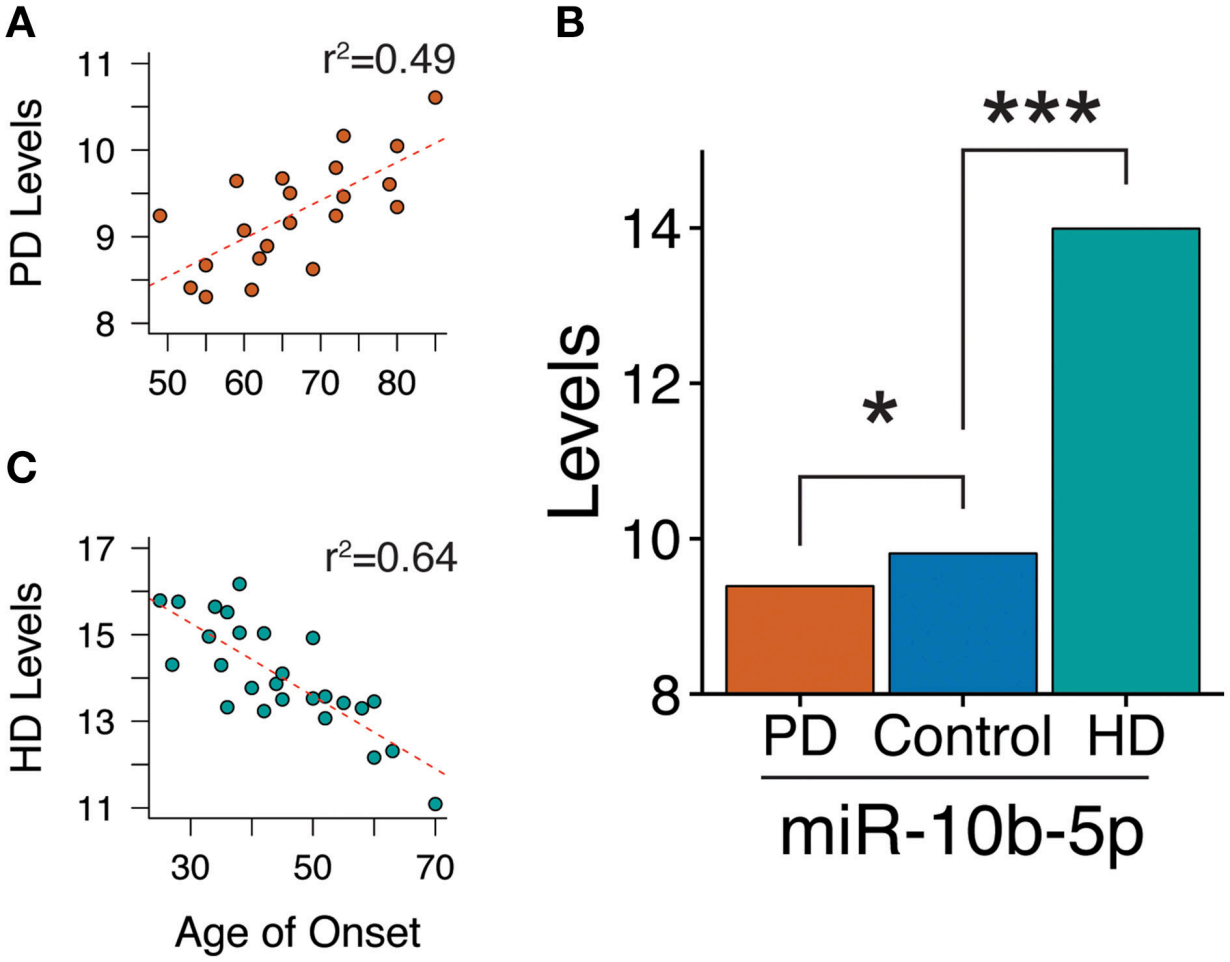
**miR-10b-5p levels are associated with motor onset age in both Parkinson's and Huntington's disease**. **(A)** Scatterplot of miR-10b-5p levels to motor onset age in Parkinson's disease (PD). In PD, miR-10b-5p levels exhibit a positive association to onset. **(B)** Comparison of miR-10b-5p level in PD and HD. ^*^q < 0.05, ^***^q < 0.001, *p*-values adjusted for genome-wide comparisons. **(C)** Scatterplot of miR-10b-5p levels to motor onset age in Huntington's disease (HD). In HD, miR-10b-5p levels exhibit a negative association to onset.

Within the PD case sample, we tested the association of age of motor onset of PD with miRNA levels. Although we did not observe significance in a genome-wide analysis, restricting our study to the 125 significantly differentially abundant PD miRNAs revealed miR-10b-5p to have a significant, positive association to onset age (beta = 0.040, *q*-value = 4.7e-2, model *r*^2^ = 0.49, see Figure 3A). Adding death age increased the magnitude of the effect estimate (death adjusted age of onset beta = 0.049, *p*-value onset = 3.2e-3, *p*-value death = 0.40), although this did not stand up to multiple comparisons corrections (*q*-value onset = 0.19). While miR-10b-5p is significantly decreased in PD, miR-10b-5p was observed in our previous HD cortical miRNA study (Hoss et al., 2014) to be massively increased in HD in comparison to controls (see Figure 3B). Intriguingly, PD and HD also exhibit opposite effects with regard to onset age, where miR-10b-5p has a strong, negative relationship to age of onset in HD (*r*^2^ = 0.64; *r*^2^ = 0.39 after accounting for the contribution of HD gene repeat length) and a strong, positive effect in PD (see Figure 3C).

## Discussion

### Parkinson's disease related miRNAs

In this study, we identified 125 miRNAs altered at genome-wide levels in PD prefrontal cortex using next-generation sequencing. This is the largest miRNA sequencing analysis performed in PD vs. control brain samples (29 vs. 33, respectively), the first to provide a detailed miRNA PD profile, classify brains by miRNA levels and to evaluate the relationship of miRNA levels in brain to relevant clinical features.

Reduced levels of miR-133b (Kim et al., 2007), miR-34b and miR-34c (Miñones-Moyano et al., 2011), and elevated levels of autophagy-related miRNAs, were previously reported (Alvarez-Erviti et al., 2013), and while these miRNAs were detectable in our study, we did not observe significant changes in their levels. These discrepancies are likely a consequence of the different brain regions that were studied (midbrain vs. prefrontal cortex), and the assay technologies that were used to profile miRNA levels (reverse transcriptase quantitative PCR Kim et al., 2007; Alvarez-Erviti et al., 2013, and microarray Miñones-Moyano et al., 2011 vs. miRNA-sequencing).

Several miRNAs that we report altered in PD brain may interact with PD-related genes. Monogenic forms of PD include mutations within the alpha-synuclein gene (*SNCA*), Leucine-rich Repeat Kinase 2 (*LRRK2*), one of the most common causes of familial PD (Nalls et al., 2014) and glucocerebrosidase (*GBA*). While we did not observe alterations of *SNCA*-targeting miRNAs, miR-7 and miR-153 (Junn et al., 2009; Doxakis, 2010), two miRNAs shown be regulated by *LRRK2* (let-7i-3p/5p and miR-184 Gehrke et al., 2010) and one miRNA experimentally shown to target *LRRK2* expression (miR-1224 Sibley et al., 2012), were observed to be down-regulated in PD.

Glucocerebrosidase (GBA) deficiency is associated with PD (Aharon-Peretz et al., 2004). We observed miR-127-5p, which has been shown to reduce GBA activity (Siebert et al., 2014), down-regulated in PD brains, and miR-16-5p which has been shown to correspond to enhanced GBA protein levels (Siebert et al., 2014), was found up-regulated in brain in our study. It is noteworthy to observe *LRRK2*-related miRNAs, as none of the PD brains in our study had *LRRK2* mutations. This may support a role of *LRRK2* and *GBA* in PD, independent of that produced by the known mutations in these genes.

### Classification based on miRNA abundance

We were able to classify PD based on the levels of 29 miRNAs with less than a 5% error rate, although this require external validation in an independent sample to confirm. While this classification was performed using postmortem brain samples, we believe this may be relevant for PD biomarker discovery, particularly if these miRNAs are peripherally detectable. We reasonably differentiated PD subtypes (PDN/PDD) based on miRNA levels, and we observed a pattern of increased changes in the PDD samples relative to the PDN samples in the set of altered miRNAs. We observed that the majority of differentially expressed miRNAs had an ordinal relationship to controls and PD cases stratified by the presence or absence of dementia, suggesting PDD may represent a more severe alteration of the PD miRNA profile.

### Circulating mirnas previously reported in PD

Our study in PD brains identified profiles of miRNAs that distinguish PD from controls, which if also observed in peripheral biofluids, such as blood or cerebrospinal fluid (CSF), could be valuable in the evaluation of PD diagnosis, prognosis, or progression. The small size (~22 nucleotides) of miRNA may allow for neuropathologically altered miRNA to cross the blood-brain barrier in exosomes (Kalani et al., 2014) and circulate stably in peripheral fluids as cell-free molecules (Mitchell et al., 2008). Although there was no overlap of miRNAs in brain to changes observed in most PD blood studies (Martins et al., 2011; Khoo et al., 2012; Cardo et al., 2013; Soreq et al., 2013; Botta-Orfila et al., 2014), we did observe increased levels in one (miR-29a-3p) of three miRNAs previously reported as increased in blood of PD patients after Levodopa treatment (Serafin et al., 2015). In Burgos et al. (2014), small RNA sequencing was performed for blood serum and CSF from 67 PD and 78 control subjects (Burgos et al., 2014), five miRNAs were found significantly altered in PD serum and 17 were significantly altered in CSF. Of these 22 miRNAs, five showed consistent overlap with our cortical findings, with one from serum (miR-1294) and four from CSF (miR-132-5p, miR-127-3p, miR-212-3p, miR-1224-5p). Thus, miRNAs detected in CSF may have a stronger relationship with brain miRNAs levels than those detected in serum.

### Common miRNA changes in PD and HD

We previously reported 75 miRNAs altered in HD prefrontal cortex (Hoss et al., 2015), and when comparing miRNAs altered in both PD and HD, 21 miRNAs were observed deregulated in both diseases. Among the miRNAs with concordant changes in PD and HD, several miRNAs correlated with various HD clinical features, such as the extent of striatal degeneration and duration of the disease (Hoss et al., 2015). These miRNAs may represent a generalized, neurodegenerative response in the prefrontal cortex, which relate to severity and/or progression across these diseases. However, it is important to recognize that the results from the PD and HD studies are not independent, as they were both analyzed using the same 33 control brains.

Of the two miRNAs with discordance between PD and HD, miR-10b-5p emerged due its relationship to onset in both diseases. miR-10b-5p is markedly increased in HD in comparison to controls, and has a negative association to age of onset for HD, with higher levels of miR-10b-5p corresponding early onset age. In contrast, miR-10b-5p is significantly decreased in PD, and has a positive association to onset age, where higher miRNA levels correspond to later onset ages. In a separate Alzheimer's disease (AD) study, examining miRNA levels in prefrontal cortex, miR-10b-5p levels were significantly reduced in AD (Lau et al., 2013). However, at the very earliest stages of AD, miR-10b-5p levels clustered with up-regulated miRNAs whereas at early to middle stages, miR-10b-5p levels appeared to decline (Lau et al., 2013). The relationship of miR-10b-5p to these three age-related, neurodegenerative diseases suggests a complicated pattern of miR-10b-5p alteration in response to the neurodegenerative or pathologic protein aggregation processes.

## Conclusion

This study provides evidence that miRNA levels are altered in PD prefrontal cortex. These changes are sufficiently consistent that diseased brains can be discriminated with high confidence from non-diseased brains based on the level of 29 miRNAs. PDD may represent a more severe profile of PD related miRNAs than PDN. 21 miRNAs changes were similar between PD and HD, with the exception of miR-10b-5p, which had opposite direction of effects to disease association and to motor onset age in the two diseases. Further characterization of miR-10b-5p in the neurodegenerative disease context is warranted to better understand if it has clinical potential as a biomarker for disease progress or to identify potential therapeutic targets.

## Author contributions

Conceived and designed the experiments: AH, JL, RM. Performed the experiments: RM, AH. Analyzed the data: AH, AL, JL. Contributed reagents/materials/analysis tools and critically reviewed the manuscript: RM, TB. Wrote the paper: AH, AL, JL, RM.

## Funding

Supported by grants from US National Institutes of Health (R01-NS076843, Characterization of the Role of Cyclin G-associated Kinase in Parkinson Disease, (R01-NS073947), Epigenetic Markers in Huntington's Disease Brain, National Science Foundation, PHY-1444389 Early-concept Grants for Exploratory Research (EAGER), U24 NS072026 National Brain and Tissue Resource for Parkinson's Disease and Related Disorders.

### Conflict of interest statement

AH, AL, JL and RM declare that the research was conducted in the absence of any commercial or financial relationships that could be construed as a potential conflict of interest. TB is a consultant for GE Healthcare and Avid Radiopharmaceuticals. The reviewer VEM and handling Editor declared a current collaboration and the handling Editor states that the process nevertheless met the standards of a fair and objective review.

## Supplementary material

Table S1 **Detailed sample information**. Missing information is designated as “NA.”

Table S2 **Results from differential expression and ordinal regression analyses**. miRNAs were removed during normalization if they failed to have an average of 2 or more counts. In PD-only analyses, some miRNAs were removed which were not removed in the analyses containing all samples. Information for undetectable miRNAs is flagged as “NA.”
